# Epitaxial growth and magnetic properties of ultraviolet transparent Ga_2_O_3_/(Ga_1−x_Fe_x_)_2_O_3_ multilayer thin films

**DOI:** 10.1038/srep25166

**Published:** 2016-04-28

**Authors:** Daoyou Guo, Yuehua An, Wei Cui, Yusong Zhi, Xiaolong Zhao, Ming Lei, Linghong Li, Peigang Li, Zhenping Wu, Weihua Tang

**Affiliations:** 1 Laboratory of Optoelectronics Materials and Devices, School of Science, Beijing University of Posts and Telecommunications, Beijing 100876, China; 2 State Key Laboratory of Information Photonics and Optical Communications, Beijing University of Posts and Telecommunications, Beijing 100876, China; 3 Center for Optoelectronics Materials and Devices, Department of Physics, Zhejiang Sci-Tech University, Hangzhou 310018, China; 4 Department of Physics, The State University of New York at Potsdam, Potsdam, New York 13676-2294, USA

## Abstract

Multilayer thin films based on the ferromagnetic and ultraviolet transparent semiconductors may be interesting because their magnetic/electronic/photonic properties can be manipulated by the high energy photons. Herein, the Ga_2_O_3_/(Ga_1−x_Fe_x_)_2_O_3_ multilayer epitaxial thin films were obtained by alternating depositing of wide band gap Ga_2_O_3_ layer and Fe ultrathin layer due to inter diffusion between two layers at high temperature using the laser molecular beam epitaxy technique. The multilayer films exhibits a preferred growth orientation of 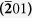 crystal plane, and the crystal lattice expands as Fe replaces Ga site. Fe ions with a mixed valence of Fe^2+^ and Fe^3+^ are stratified distributed in the film and exhibit obvious agglomerated areas. The multilayer films only show a sharp absorption edge at about 250 nm, indicating a high transparency for ultraviolet light. What’s more, the Ga_2_O_3_/(Ga_1−x_Fe_x_)_2_O_3_ multilayer epitaxial thin films also exhibits room temperature ferromagnetism deriving from the Fe doping Ga_2_O_3_.

During the past ten years, multilayer thin films based on magnetic and nonmagnetic layers have great deal of scientific and industrial attention due to their anomalous spin dependent effects and potential applications in magnetic sensors, information storage media, thermoelectric devices and high frequency devices^1^,^2^,^3^. Particularly, the ferromagnetic and transparent semiconductors multilayer thin films are interesting because where the ferromagnetic properties can be controlled by light^4^,^5^,^6^. It integrates magnetism into optoelectronics devices, appealing materials for magnetooptical devices^7^,^8^. So far, there have been a few reports on transparent ferromagnetic multilayer thin films. For example, ZnO/Fe_3_O_4_ and In_2_O_3_/Fe_3_O_4_ multilayer structure films were fabricated and their optoelectrical and magnetic properties were studied^9^,^10^,^11^. As a typical wide band gap semiconductor material, *β-*Ga_2_O_3_, with a band gap of 4.5~5.3 eV and a high transparency for the visible and wide range of ultraviolet down to 280 nm^12^,^13^,^14^, is considered as one of ideal candidates to fabricate transparent multilayer magnetic-optic-electronic devices^15^,^16^. Ga_2_O_3_ can crystallize in five different phases (*α*, *β*, *γ*, *δ*, and *ε*)^17^,^18^. Amongst them, the monoclinic *β*-Ga_2_O_3_ (space group: C2/m) with the lattice parameters of *a* = 12.23 Å, *b* = 3.04 Å, *c* = 5.80 Å, and *β* = 103.7° is considered to be the most stable thermally in the range from room temperature up to the melting point of about 1800 °C what determines also the possibility of working at high temperatures^19^. Additionally, *β*-Ga_2_O_3_ has great chemical stability, being unaffected even by concentrated acids such as hydrofluoric acid^20^. More importantly, *β*-Ga_2_O_3_ is known to exhibit a strong dynamic nuclear polarization upon saturation of the magnetic resonance of conduction electrons, which is at the origin of a free electron memory referred to as bistable conduction electron spin resonance^21^,^22^. On the other hand, *β*-Ga_2_O_3_ is also the host material for magnetic semiconductors, and room temperature ferromagnetism was observed in Mn-doped *β*-Ga_2_O_3_ by our group^15^.

The *β*-Ga_2_O_3_ and *β*-Ga_2_O_3_ based magnetic semiconductors multilayer thin films with a high ultraviolet transparency may exhibit some special magnetic/electronic/photonic properties through the manipulation of high energy photons. Generally, epitaxial growth is very essential for the properties of these multilayer thin films. Recent improvements in deposition technology enable an alternative approach in the manipulation of ultrathin layer for growing multilayer thin films. Herein, we fabricated the Ga_2_O_3_/(Ga_1−x_Fe_x_)_2_O_3_ multilayer epitaxial thin films and investigated their structural, optical, and magnetic properties.

## Results and Discussion

**Figure 1:**
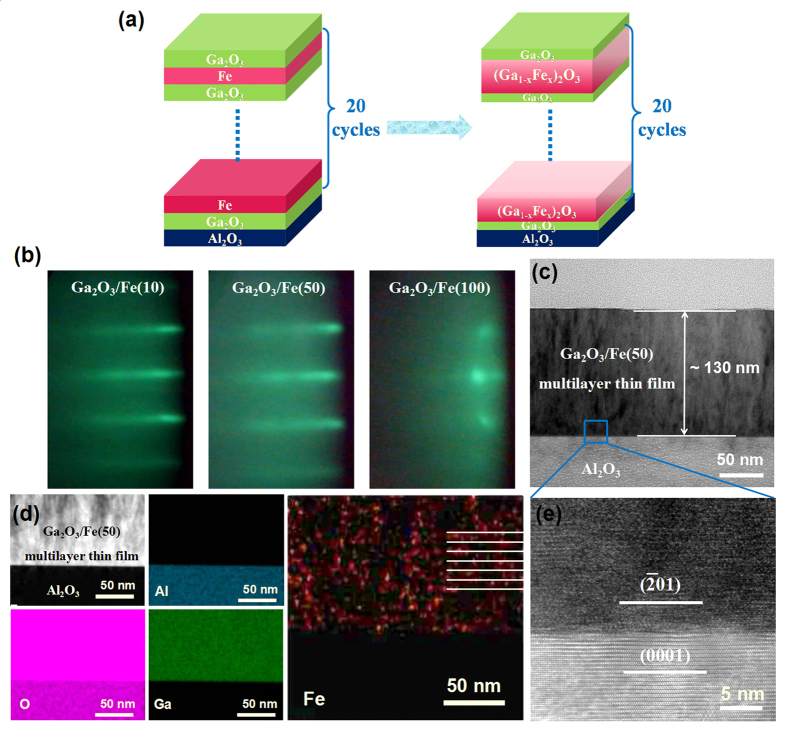
(**a**) Schematic diagram of alternating deposition of Ga_2_O_3_ and Fe ultrathin layers for fabricating the Ga_2_O_3_/(Ga_1−x_Fe_x_)_2_O_3_ multilayer thin films; (**b**) RHEED patterns of the Ga_2_O_3_/Fe(10), Ga_2_O_3_/Fe(50) and Ga_2_O_3_/Fe(100) multilayer thin films; (**c**) Cross-sectional low-magnification TEM bright-field image of the Ga_2_O_3_/Fe(50) multilayer thin film; (**d**) TEM-EDX measurement of cross-sectional observation image of the interface between Ga_2_O_3_/Fe(50) multilayer thin film and Al_2_O_3_ substrate, and the composition distributions of Al, O, Ga and Fe elements drew by different colors; (**e**) Cross-sectional high-resolution TEM image of the interface between the Ga_2_O_3_/Fe(50) multilayer film and Al_2_O_3_ substrate as marked by a blue small pane in (**c**).

The Ga_2_O_3_/(Ga_1−x_Fe_x_)_2_O_3_ multilayer thin films were fabricated by alternating depositing of Ga_2_O_3_ layer and Fe ultrathin layer due to inter diffusion between two layers at high temperature using the laser molecular beam epitaxy (LMBE) technique, as is shown in Fig. 1(a). The thickness and composition of (Ga_1−x_Fe_x_)_2_O_3_ thin layer can be controlled by solely changing the laser pulse numbers during each run for depositing the Fe layer (defined as N, N = 0, 10, 20, 30, 40, 50,100) while those for depositing Ga_2_O_3_ layers in each run were fixed at 100. Herein, samples were abbreviated as Ga_2_O_3_/Fe(N) multilayer thin films for the laser pulse numbers N of depositing Fe layer. The sharp and streaky reflection high-energy electron diffraction (RHEED) patterns indicate the achievement of an atomically flat surface of Ga_2_O_3_/Fe(10) and Ga_2_O_3_/Fe(40) multilayer thin films [Fig. 1(b)]. While the RHEED patterns of Ga_2_O_3_/Fe(100) film are fuzzy and punctiform, meaning a rough surface. Cross-section low-magnification transmission electron microscope (TEM) investigations were carried out to identify the microstructure of the thin film. The Ga_2_O_3_/Fe(50) multilayer thin film shows a thickness of about 130 nm estimated by the cross-sectional TEM bright-field image of Fig. 1(c). The average thickness of the Ga_2_O_3_/Fe(50) single thin layer is about 6.5 nm due to the layer number of 20. Figure 1(e) shows the cross-sectional high-resolution TEM image of the interface between the Ga_2_O_3_/Fe(50) multilayer film and Al_2_O_3_ substrate as marked by a blue small pane in Fig. 1(c), which clearly indicates the orientation relationship of 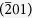 *β*-Ga_2_O_3_//(0001) Al_2_O_3_. However, it does not found obvious multilayer structure, which can be attributed to the following two factors: (1) The radii of Fe and Ga cations are close (Fe^2+^, Fe^3+^, and Ga^3+^ ionic are 0.74, 0.64, and 0.62 Å, respectively^23^,^24^,^25^), and the lattice parameters of Ga_2_O_3_ change rarely with the Fe doping; (2) The Fe doping concentration of the Ga_2_O_3_/Fe(N) multilayer thin film is low (it is 2.44 at.% for N = 50). To investigate the composition distributions in the Ga_2_O_3_/Fe multilayer thin films, the elemental composition mapping of cross-sectional observation was obtained by the energy dispersive X-ray (EDX) measurement in TEM. The elements of Ga, Fe, Al, and O which compose Ga_2_O_3_/Fe(50) and Al_2_O_3_ were chose as the target elements, as is shown in Fig. 1(d). From the analysis results, Ga and O elements are distributed uniformly and there are no remarkable phase separating area in the Ga_2_O_3_/Fe(50) film. However, Fe element exhibits clear agglomerated areas and obvious stratified phenomenon. Al metal is derived from the Al_2_O_3_ substrate and O is from thin film and substrate.

**Figure 2:**
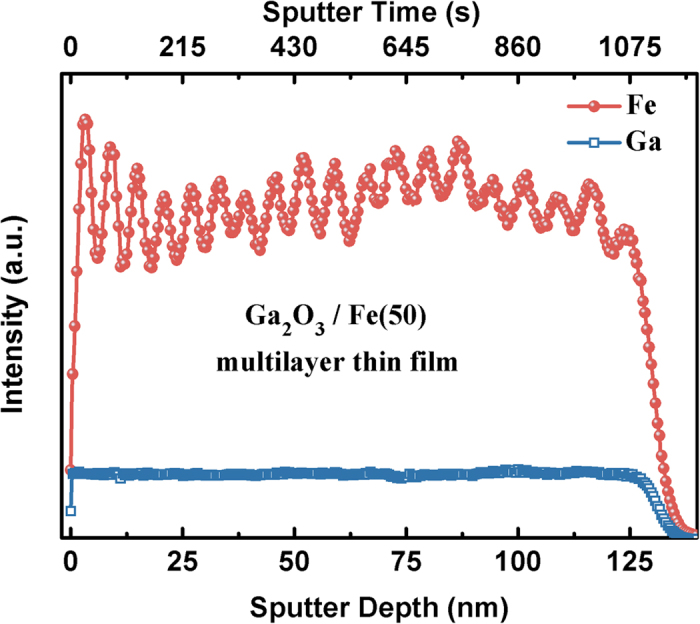
Fe and Ga SIMS depth profiles for the Ga_2_O_3_/Fe(50) multilayer thin film.

In order to further confirm the multilayer structure of the as-grown thin films, the compositions as a function of film thickness were characterized by using the secondary ion mass spectrometry (SIMS) depth profiling. The results for the representative Ga_2_O_3_/Fe(50) film were given in Fig. 2 by showing intensities of the Fe and Ga ion currents as a function of sputter depth of the film. The intensity of Ga ion remains almost constant as the sputter depth of the film. However, the intensity of Fe ion exhibits evident wavy shape with 19 peaks, corresponding to the 19 layers of (Ga_1−x_Fe_x_)_2_O_3_ thin film in our scheming Ga_2_O_3_/(Ga_1−x_Fe_x_)_2_O_3_ multilayer structure. Notably, the difference value between peaks and troughs of few layers near-surface is larger than that near-substrate, which is attributed to the inter diffusion between Fe ultrathin layer and Ga_2_O_3_ layer at high temperature for much more time. As a result, both the TEM-EDX and SIMS results indicate that Fe ion is uneven and stratified distributed in the film.

**Figure 3:**
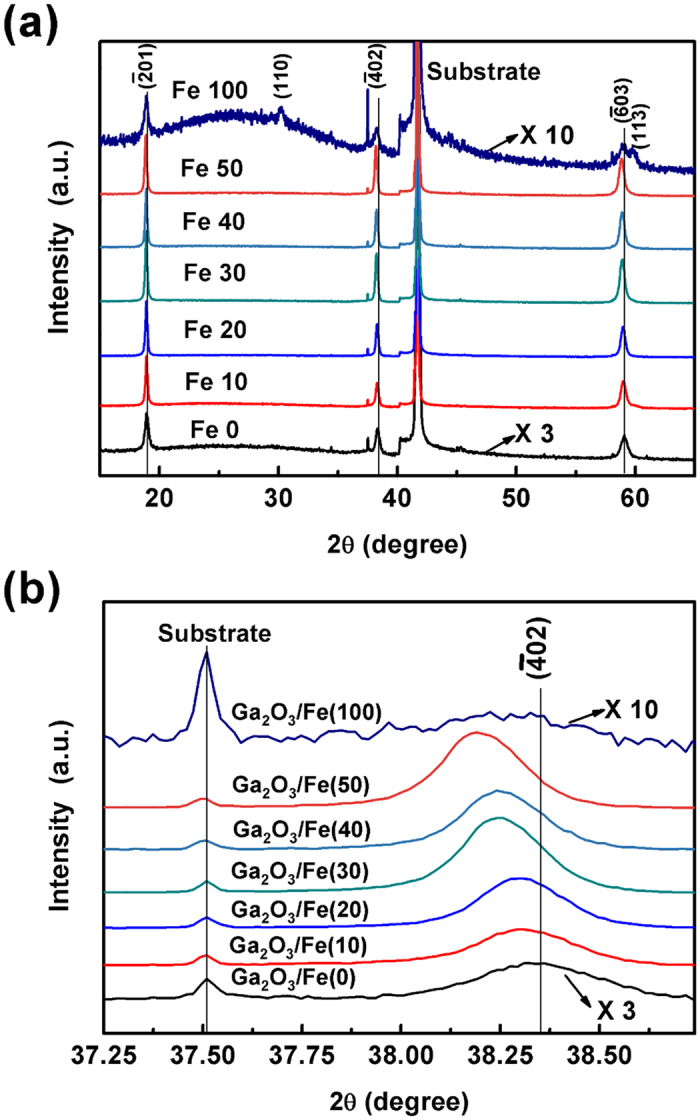
(**a**) θ–2θ XRD patterns of the Ga_2_O_3_/Fe(N) multilayer thin film; (**b**) Enlarged view of θ–2θ XRD patterns around 38°.

The crystal structure and film orientation of the Ga_2_O_3_/(Ga_1−x_Fe_x_)_2_O_3_ multilayer thin films were determined from θ–2θ scans of XRD, as is shown in Fig. 3(a). In order to facilitate compared with the others, the diffraction intensities of the Ga_2_O_3_/Fe(0) and Ga_2_O_3_/Fe(100) films were multiplied by 3 and 10 times respectively. Except Ga_2_O_3_/Fe(100) film, only 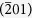 and higher order peaks of Ga_2_O_3_ monoclinic *β* phase appear for the other as-grown Ga_2_O_3_/(Ga_1−x_Fe_x_)_2_O_3_ multilayer films besides those from the substrate, indicating single phase and a preferred 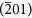 plane orientation of the films. Furthermore, as shown in Fig. 3(b) with the enlarged view of θ–2θ XRD patterns around 38°, the peaks of 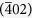 are located at 38.36°, 38.31°, 38.29°, 38.26°, 38.24°, and 38.19° for N = 0, 10, 20, 30, 40, and 50, respectively, indicating that the peak gradually shifts to smaller 2θ with the increase of Fe layer thickness. The lower angles shift of 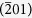 and higher order diffraction peaks indicates an increase of the lattice constants, which means that Fe ion has incorporated into Ga ion site and possesses a larger radius than Ga ion^23^,^24^,^25^. However, for the Ga_2_O_3_/Fe(100) film, extra diffraction peaks of (110) and (113) of *β* phase Ga_2_O_3_ appear and the diffraction intensity of the family of 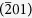 crystal planes decreases, featuring a polycrystalline nature. No impurity peaks related to Fe metal clusters and Fe oxides were observed.

**Figure 4:**
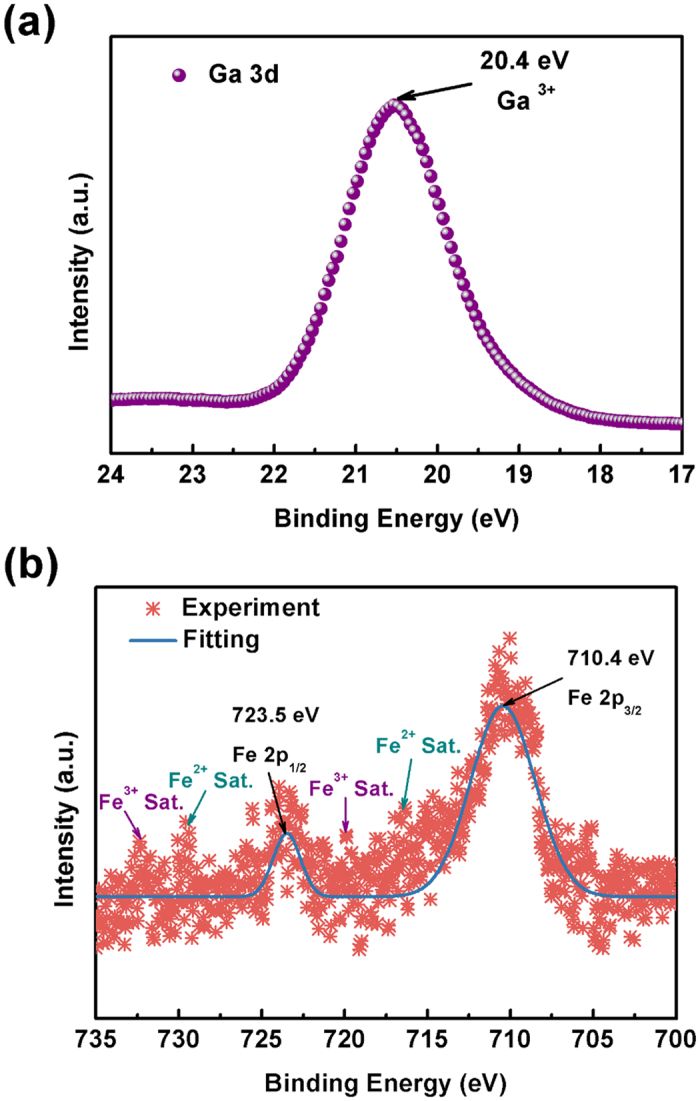
XPS spectra of Ga 3d (**a**) and Mn 2p (**b**) core level for the Ga_2_O_3_/Fe(50) multilayer thin film.

The chemical compositions and chemical states of Fe ions in the as-grown films were characterized by using X-ray photoelectron spectroscopy (XPS), presented in Fig. 4. The elements present in the Ga_2_O_3_/Fe(50) multilayer film are Fe, Ga, O, and C (not shown). The reason of C found in the samples was asserted to be due to surface contamination. The charge-shift spectrum was calibrated using the fortuitous C 1s peak at 284.8 eV. The energy peak for Ga 3d is centered at 20.4 eV [Fig. 4(a)], which is attributable to the presence of Ga-O bond^26^. The high-resolution XPS spectrum of Fe 2p core level shows a spin-orbit doublet (j = 3/2, 1/2) [Fig. 4(b)]. Of the two peaks Fe 2p_3/2_ peak is narrower and stronger than Fe 2p_1/2_ and the area of Fe 2p_3/2_ peak is greater than that of Fe 2p_1/2_ because Fe 2p_3/2_ has degeneracy of four states whilst Fe 2p_1/2_ has only two. For Fe^2+^ cations, the peak position of Fe 2p_3/2_ and Fe 2p_1/2_ is located at about 709.0 and 722.6 eV, and the satellite peak of Fe 2p_3/2_ is located approximately 6 eV higher than the main Fe 2p_3/2_ peak; While for Fe^3+^ cations, the peak position of Fe 2p_3/2_ and Fe 2p_1/2_ is located at about 711.0 and 724.6 eV, and the satellite peak of Fe 2p_3/2_ is located approximately 8 eV higher than the main Fe 2p_3/2_ peak^27^,^28^. In our Ga_2_O_3_/(Ga_1−x_Fe_x_)_2_O_3_ multilayer thin films, the peak positions of Fe 2p_3/2_ and Fe 2p_1/2_ are, respectively, 710.4 and 723.5 eV. They are located between the values for Fe^2+^ and Fe^3+^ cations, which can be deconvoluted into the Fe^2+^ and Fe^3+^ peaks, indicating that both Fe^2+^ and Fe^3+^ cations are contained. In addition, the satellite peaks at the high binding energy side of the main peaks are further demonstrated the coexistence of Fe^2+^ and Fe^3+^.

**Figure 5:**
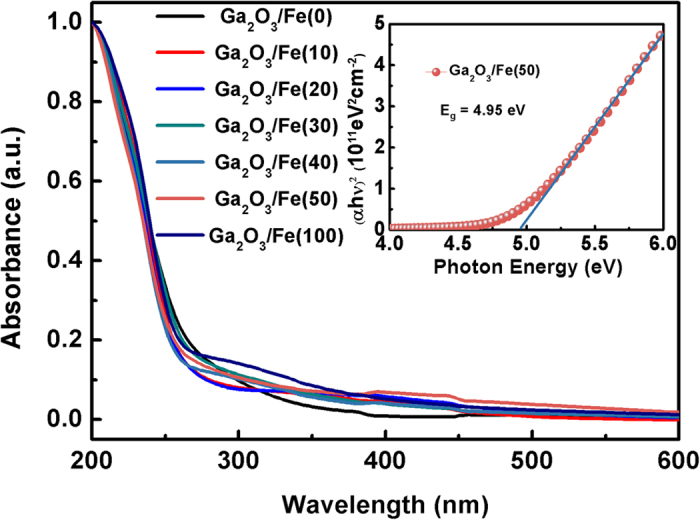
Absorption spectra of the Ga_2_O_3_/Fe(N) multilayer thin films and the plot of (*αhν*)^2^ versus *hν* for the Ga_2_O_3_/Fe(50) film (inset).

Figure 5 shows the ultraviolet-visible (UV-Vis) absorbance of the Ga_2_O_3_/(Ga_1−x_Fe_x_)_2_O_3_ multilayer thin films. All the samples exhibit a sharp absorption edge at about 250 nm, corresponding to the intrinsic absorption of *β*-Ga_2_O_3_^13^,^14^. The absorption onset shows no obvious shift with the increase of Fe ultrathin layer thickness. The band gap is derived by fitting the linear region of the plot (*αhν*)^2^ versus *hν*. The inset of Fig. 5 shows the Ga_2_O_3_/Fe(50) multilayer film has a band gap of about 4.95 eV. On the other hand, it is observed that all the Ga_2_O_3_/(Ga_1−x_Fe_x_)_2_O_3_ multilayer films has no obvious absorbance to the wavelength until 250 nm, indicating a high transparency for ultraviolet light.

**Figure 6:**
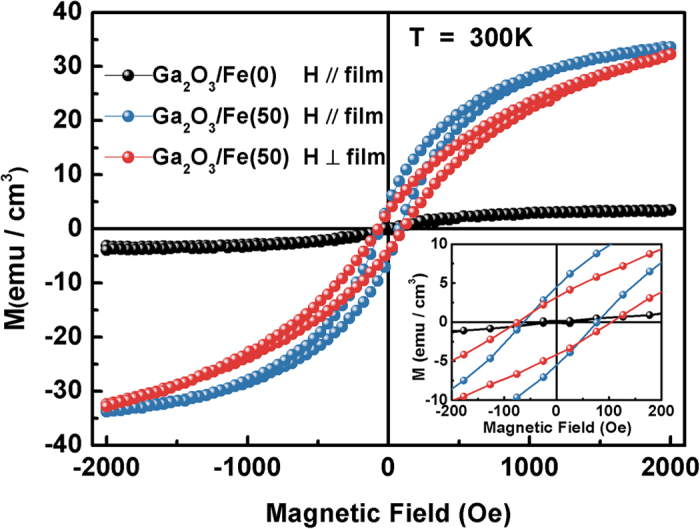
Magnetic field dependence of magnetization (*M-H* curve) and the corresponding enlarged image (inset) of the Ga_2_O_3_/Fe(50) multilayer thin film as the magnetic field is parallel and perpendicular to the film compared with that of pure *β*-Ga_2_O_3_ thin film.

Figure 6 shows the magnetization versus magnetic field (*M-H*) curves of the Ga_2_O_3_/Fe(50) multilayer thin film at room temperature with that of pure *β*-Ga_2_O_3_ thin film for comparing. The diamagnetic contribution from the *α*-Al_2_O_3_ substrate was subtracted from the data. The pure *β*-Ga_2_O_3_ thin film [Ga_2_O_3_/Fe(0)] displays paramagnetic behavior while Ga_2_O_3_/Fe(50) film show hysteresis loops indicative of ferromagnetism when the applied magnetic field is parallel to the films. Furthermore, the Ga_2_O_3_/Fe(50) multilayer thin film exhibits a magnetic anisotropy while applying the magnetic field parallel and perpendicular to the film surface. As seen in the enlarged image of *M-H* loops in the inset of Fig. 6, the coercivity and magnetic remanence (*M*_*r*_) are ~73 Oe and 4.99 emu/cm^3^ for the magnetic field paralleling to the film, while they are ~91 Oe and 3.68 emu/cm^3^ respectively for the perpendicular one. And the saturation magnetization (*M*_*s*_) of the Ga_2_O_3_/Fe(50) multilayer is similar for parallel and perpendicular which are 33.8 and 32.8 emu/cm^3^ respectively at 2 T. The magnetic anisotropy in Ga_2_O_3_ with doping other transition metal (such as Mn, Cr) has also been reported^15^,^16^. Origin of the anisotropic magnetization is not clear at the moment. The anisotropic behavior cannot be explained by the presence of randomly oriented ferromagnetic particles. In addition, the XRD and XPS measurements have confirmed the successful substitution of Fe for Ga and ruled out the possible secondary phases of Fe metal cluster and Fe-based oxides. It is reasonably to conclude that the room temperature ferromagnetism in the Ga_2_O_3_/(Ga_1−x_Fe_x_)_2_O_3_ multilayer films is intrinsic. The perpendicular & parallel magnetic moments of Fe cation in the Ga_2_O_3_/Fe(50) multilayer thin film at 2 T are 3.74 and 3.89 *μ*_B_/Fe cation respectively, which are very close to the magnetic moment of Fe cation reported by others^29^,^30^.

In conclusion, the multilayer epitaxial thin films based on wide band gap of Ga_2_O_3_ and magnetic semiconductor of (Ga_1−x_Fe_x_)_2_O_3_ were fabricated by alternating depositing of Ga_2_O_3_ layer and Fe ultrathin layer by LMBE. The systematic characterizations by XRD, TEM-EDX, SIMS, XPS and UV-Vis absorbance spectrum confirmed the incorporation of Fe into the lattice of *β-*Ga_2_O_3_ and the formation of the Ga_2_O_3_/(Ga_1−x_Fe_x_)_2_O_3_ multilayer epitaxial thin films. Optical and magnetic properties measurements revealed that the multilayer films are high ultraviolet transparency and room temperature ferromagnetism.

## Methods

The epitaxial thin films were prepared on 10 × 10 mm *α*-Al_2_O_3_ (0001) substrates by the LMBE technique at a repetition frequency of 1 Hz and with a fluence of ~5 J/cm^2^. The thin film deposition was grown in a vacuum environment of 1 × 10^−6^ Pa and at a substrate temperature of 900 °C. Alternating depositions of Ga_2_O_3_ layer and Fe ultrathin layer were performed for 20 times to prepare the Ga_2_O_3_/(Ga_1−x_Fe_x_)_2_O_3_ multilayer epitaxial thin films. The (Ga_1−x_Fe_x_)_2_O_3_ layer (that is Fe doping Ga_2_O_3_ layer) was obtained due to inter diffusion between Fe and Ga_2_O_3_ layers at high temperature. The Fe doping concentration of the Ga_2_O_3_/Fe(50) multilayer thin film was determined as 2.44 at.% by the X-ray energy dispersive spectroscopy. The targets were kept inside the chamber, so that deposition of all the layers could be done without breaking vacuum. This is essential to avoid any contaminations of interfaces. RHEED was utilized *in-situ* to monitor the whole epitaxial growth process. The orientation and crystallinity of the as-grown thin films were investigated by the XRD at θ–2θ scan. The thickness and microstructure of thin films were obtained by the TEM. Cross-section TEM specimens were prepared by a standard procedure which includes mechanical grinding, polishing, precision dimpling, and ion milling. The valences of Mn ions and elements distribution were analyzed by XPS and SIMS. Magnetic properties of the films were measured in a commercial superconducting quantum interference device (SQUID), Quantum design.

## Additional Information

**How to cite this article**: Guo, D. *et al.* Epitaxial growth and magnetic properties of ultraviolet transparent Ga_2_O_3_/(Ga_1−x_Fe_x_)_2_O_3_ multilayer thin films. *Sci. Rep.* **6**, 25166; doi: 10.1038/srep25166 (2016).
